# Supplementary material for: On the elephant trails: habitat suitability and connectivity for Asian elephants in eastern Indian landscape
Source: PeerJ. 2024 Mar 29;12:e16746. doi: 10.7717/peerj.16746 (PMC10984178; doi:10.7717/peerj.16746)
Supplement: Supplemental Information 2 [file peerj-12-16746-s002.docx]

**Supplementary Figure:**


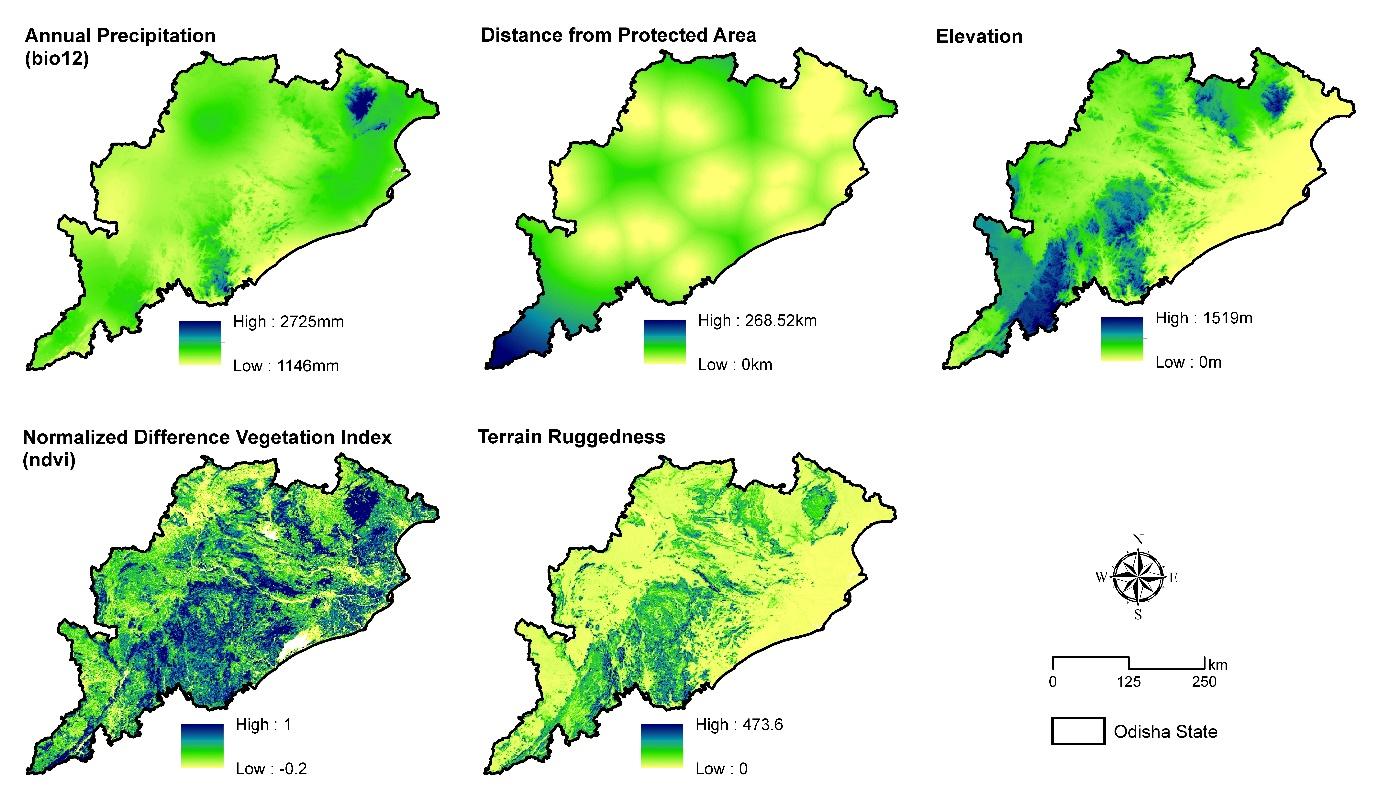


*Supp. Figure 1: Non-correlated predictor variables used to model the potential habitat use and movement areas for Asian elephants in Odisha, India. The map was generated using ArcMap version 10.8 (www.support.esri.com).*

**Supplementary Tables:**

*Supp. Table 1: Predictor variables used for ensemble model, their spatial information, sources and hypothesis for the elephant habitat suitability in Odisha, India*

| **Predictors** | **Mean ± SD (min-max) unit** | **Spatial resolution** | **Source/Reference** |
| --- | --- | --- | --- |
| Normalized Difference Vegetation Index (NDVI) | 0.75±0.12  (-0.2-1) | 1 km | AppEEARS NASA erath data portal (www.appeeats.earthdatacloud.nasa.gov) |
| Terrain ruggedness | 51.89±61.31 (0-473.63) | 1 km | Riley et al., 1999 (derived from elevation) |
| Elevation | 305.4±253.34 (0-1519.51) m | 1 km | USGS Earth explorer (www.earthexplorer.usgs.gov) |
| Distance from protected area | 43.94±41.28 (0-268.52) km | 1 km | GeoNode portal (www.geo.ejatlas.org) |
| Annual precipitation | 1465.2±131.83 (1146-2725) mm | 1 km | Fick and Hijmans, 2017 (www.worldclim.org) |

*Supp. Table 2: List of selected forest areas as core habitat nodes for modelling the potential movement areas for Asian elephants in Odisha, India*

| **Node ID** | **Name of core habitat node** | **Area (km^2^)** | **List of protected areas and reserve forest (RF) and protected forest (PF) under the core habitat node** |
| --- | --- | --- | --- |
| 1 | Similipal | 2750 | Similipal TR, Mahubhandar RF, Kanthai RF, Muludihi RF, Bispur RF, Kendumundi RF, Salaibera RF, Cherratangar RF, Dangapani RF, Kadopani RF, Ghorabindha RF, Dangadha RF, Mankarbera RF, Bhejdiha RF, Gorumahisani RF, Jari RF, Sarali RF and nearby forest patches |
| 2 | Jada | 905.54 | Kumaria RF, Rutkupiri RF, Sagjor RF, Jambua RF, Khinda RF, Bhainsamuna RF, Sirgida RF, Kusmdihi RF, Balai RF, Jada RF, Kunjar RF, Kantamunda RF, Betjharan RF, Rajudima block RF, Raipiri RF, Kelo RF |
| 3 | Siddhamath | 341.46 | Uliburu RF, Thakurani RF, Karu RF, Siddhamath RF, Lakrhaghat RF, Karo RF, Baitarani RF |
| 4 | Garjanpahar | 431.88 | Garjanpahar RF, Dhanubansh RF, Jamiharha PF, Kharudaldali PF, Korbahal PF, Kanthidungri PF, Chhengapahar RF, Barghumra RF, Singaribahal RF, Rohni RF, Dhenkapani RF, Barbahali RF, Budlakhaman RF, Julambahal PF, Chitkikharu RF, Sukhadihi RF, Jhargan RF, Bhanwarkhol RF, Bikramkhol RF, Telia PF, Kaudhara PF |
| 5 | Debrigarh | 423.69 | Debrigarh RF, Lohara RF, Dechua RF |
| 6 | Lakhari valley | 196.27 | Lakhari Valley WLS, Chadragiri RF |
| 7 | Khalasuni | 696.55 | Badarma WLS, Khalasuni WLS and RF, Ushakothi RF, Salguni RF |
| 8 | Anantapur | 234.45 | Bulajhar RF, Anantapur RF, Jiridamali RF, Nandi RF, Madhi RF |
| 9 | Kapilash | 317.87 | Kapilash WLS and RF, Gondia RF, Khankira RF, Ramai RF, Goinda RF, Sathlundi RF, Karanji RF, Baniabandha RF, Ambiljhari RF, Dalijora RF, Patapuri RF, Gobra RF, Ranibania RF, Gadabola RF, Adala RF |
| 10 | Satkosia | 1440.2 | Baisipalli WLS and RF, Satkosia Gorge WLS, Hatidhara RF, Baghmunda RF, Tainsi RF, Kumuri RF, Kurru RF, Maihipara RF, Purnakot RF, Raigorha RF, Tulka RF, Tikarapara RF, Padmatala RF, Arakhpadar RF, Mahanadi RF |
| 11 | Subarnagir | 474.2 | Bahabalakhol RF, Penajura RF, Subarnagir RF, Rabaneshwar RF, Matakupa RF, Kalabag RF, Palacmi RF |
| 12 | Sunabeda | 1256.45 | Sunabeda WLS, Lodra RF, Ranimai PF, Manikgarh PF |
| 13 | Hattigam | 115.47 | Hattigam RF |
| 14 | Karlapat | 171.58 | Karlapat WLS and RF, Nehela RF, Shagarha RF, Jugasaipatna RF, Hatimunda RF, Khalia RF, Jerka RF |
| 15 | Kothagarh | 699.53 | Kothagarh WLS, Madagurha RF, Guma RF, Haripur RF, Kilang block RF, Laseri RF, Subarnagiri RF, Shriramapur RF, Bandaru RF, Kesharigurha RF, Durgapanga RF, Hanumantapur RF, Ambimaha RF |
| 16 | Barabara | 868.53 | Maninaga RF, Patharaganda RF, Badamal RF, Banijhari RF, Gochha RF, Dhanai RF, Patia RF, Tamna RF, Arang RF, Rajin RF, Sulia RF, Sandhamul RF, Chakarapathar RF, Nathpur RF, Kariaba RF, Kadambajhor RF, Jeripara RF, Latwal RF, Pandiriparha RF, Bhatapara RF, Chulijhinka RF, Kumaripari Boraib RF, Jokalandi RF |
| 17 | Dharamagarh | 541.99 | Sindhigan RF, Dandarkhol RF, Tentuli RF, Dharamagarh RF, Dharmagurha RF, Tulasi RF, Sarangapalli RF, Dasmantapur RF |
| 18 | Chandaka | 182.68 | Chandaka WLS, Damaparha RF, Dalua RF, Bhola RF, Koduamunda RF, Haladia RF, Tarakai RF, Banatala RF |
| 19 | Kuldiha | 232.83 | Kuldiha WLS, Tenda RF, Debagiri RF, Garasahi RF, Khuntapatana RF |

*Supp. Table 3. Predictive abilities of five modelling algorithms used to estimate potential habitat use by Asian elephants in Odisha, India*

| **Modelling algorithm** | **AUC (ROC) ± SD** | **TSS ± SD** | **KAPPA ± SD** |
| --- | --- | --- | --- |
| **Generalized linear model (GLM)** | 0.76 ±0.006 | 0.44 ± 0.014 | 0.4 ± 0.013 |
| **Gradient boosted machine (GBM)** | 0.79 ±0.004 | 0.47 ± 0.006 | 0.42 ± 0.009 |
| **Generalized additive model (GAM)** | 0.79 ±0.006 | 0.46 ± 0.011 | 0.41 ± 0.01 |
| **Multiple adaptive regression splines (MARS)** | 0.79 ±0.004 | 0.46 ± 0.011 | 0.41 ± 0.007 |
| **Random forest (RF)** | 0.81 ±0.003 | 0.51 ± 0.009 | 0.46 ± 0.009 |
| **Maximum entropy (MaxEnt)** | 0.79 ±0.004 | 0.46 ± 0.011 | 0.41 ± 0.011 |
| **Ensemble Model** | 0.94 | 0.71 | 0.73 |

*Abbreviations:* AUC: Area under the receiver operating characteristic curve; TSS: True skill statistics; SD: Standard Deviation

*Supp. Table 4: Relative contributions of the five selected predictor variables in the ensemble model of potential habitat use by Asian elephants in Odisha, India*

| **Predictor variable** | **Variable Contribution in Ensemble Model** |
| --- | --- |
| **Normalized difference vegetation index (NDVI)** | 0.42 ± 0.004 |
| **Terrain Ruggedness** | 0.19 ± 0.001 |
| **Elevation** | 0.17 ± 0.002 |
| **Distance from protected area** | 0.13 ± 0.003 |
| **Annual precipitation (bio12)** | 0.08 ± 0.002 |

*Supp. Table 5: Details on forest and wildlife divisions-wise potential habitat use by Asian elephants in the state of Odisha, India*

| Forest & Wildlife Divisions | Serial No. (as mentioned in Fig.3) | Highly potential | Moderate highly potential | Moderately potential | Moderate least potential | Least potential |
| --- | --- | --- | --- | --- | --- | --- |
| Sundargarh Forest Division | 1 | 625.27 (15.95%) | 807.23 (20.59%) | 569.03 (14.51%) | 826.25 (21.07%) | 1093.4 (27.88%) |
| Sunabeda Wildlife Division | 2 | 144.74 (16.79%) | 426.77 (49.52%) | 208.42 (24.18%) | 72.78 (8.45%) | 9.1 (1.06%) |
| STR Wildlife Division | 3 | 401.13 (44.05%) | 381.28 (41.87%) | 122.41 (13.44%) | 5.79 (0.64%) | 0 (0%) |
| Subarnapur Forest Division | 4 | 238.2 (10.08%) | 179.48 (7.59%) | 178.65 (7.56%) | 402.79 (17.04%) | 1364.68 (57.73%) |
| Satkosia Wildlife Division | 5 | 368.05 (57.94%) | 148.05 (23.31%) | 38.05 (5.99%) | 34.74 (5.47%) | 46.32 (7.29%) |
| Hirakud Wildlife Division | 6 | 136.47 (14.21%) | 156.32 (16.28%) | 56.24 (5.86%) | 46.32 (4.82%) | 564.89 (58.83%) |
| Sambalpur South Forest Division | 7 | 544.22 (25.96%) | 255.57 (12.19%) | 172.03 (8.21%) | 390.38 (18.62%) | 734.44 (35.03%) |
| Sambalpur North Forest Division | 8 | 128.2 (7.72%) | 157.14 (9.46%) | 139.78 (8.41%) | 379.63 (22.85%) | 856.85 (51.57%) |
| Rourkela Forest Division | 9 | 633.54 (21.38%) | 268.8 (9.07%) | 231.58 (7.82%) | 473.92 (16%) | 1354.75 (45.73%) |
| Rayagada Forest Division | 10 | 938.73 (12.8%) | 2097.47 (28.59%) | 2048.67 (27.93%) | 1423.4 (19.4%) | 827.9 (11.29%) |
| Rajnagar Wildlife Division | 11 | 0 (0%) | 0 (0%) | 0.83 (0.03%) | 7.44 (0.3%) | 2465.52 (99.67%) |
| Rairangpur Forest Division | 12 | 557.45 (23.17%) | 438.35 (18.22%) | 255.57 (10.62%) | 302.71 (12.58%) | 851.89 (35.41%) |
| Rairakhol Forest Division | 13 | 1104.97 (54.42%) | 346.55 (17.07%) | 179.48 (8.84%) | 215.87 (10.63%) | 183.61 (9.04%) |
| Chilika Wildlife Division | 14 | 0.83 (0.36%) | 4.96 (2.16%) | 11.58 (5.05%) | 22.33 (9.75%) | 189.4 (82.67%) |
| Puri Wildlife Division | 15 | 0 (0%) | 0 (0%) | 0 (0%) | 4.96 (0.3%) | 1674 (99.7%) |
| Phulbani Forest Division | 16 | 420.98 (11.79%) | 676.55 (18.94%) | 880.84 (24.66%) | 1219.11 (34.14%) | 373.84 (10.47%) |
| Nayagarh Forest Division | 17 | 873.39 (28.67%) | 593.84 (19.49%) | 389.55 (12.79%) | 371.36 (12.19%) | 817.98 (26.85%) |
| Nawarangpur Forest Division | 18 | 19.85 (0.39%) | 103.38 (2.04%) | 388.73 (7.68%) | 970.99 (19.19%) | 3577.11 (70.69%) |
| Malkangiri Forest Division | 19 | 0 (0%) | 0 (0%) | 0 (0%) | 0 (0%) | 5528.18 (100%) |
| Mahanadi Wildlife Division | 20 | 199.33 (48.01%) | 133.16 (32.07%) | 36.39 (8.76%) | 23.16 (5.58%) | 23.16 (5.58%) |
| Koraput Forest Division | 21 | 86.02 (1.66%) | 282.86 (5.46%) | 405.27 (7.83%) | 727.83 (14.06%) | 3675.53 (70.99%) |
| Khurda Forest Division | 22 | 415.19 (11.01%) | 217.52 (5.77%) | 263.01 (6.97%) | 392.03 (10.39%) | 2483.71 (65.86%) |
| Khariar Forest division | 23 | 36.39 (1.23%) | 263.84 (8.9%) | 387.07 (13.05%) | 613.69 (20.7%) | 1664.08 (56.12%) |
| Keonjhar Wildlife Division | 24 | 601.28 (29.71%) | 325.87 (16.1%) | 243.16 (12.01%) | 199.33 (9.85%) | 654.22 (32.33%) |
| Keonjhar Forest Division | 25 | 1122.34 (18.14%) | 1244.75 (20.12%) | 1147.98 (18.55%) | 1427.53 (23.07%) | 1244.75 (20.12%) |
| Karanjia Forest Division | 26 | 436.7 (19.57%) | 630.23 (28.24%) | 442.49 (19.83%) | 339.1 (15.2%) | 382.94 (17.16%) |
| Kalahandi South Forest Division | 27 | 436.7 (10.84%) | 787.38 (19.54%) | 513.61 (12.75%) | 625.27 (15.52%) | 1665.73 (41.35%) |
| Kalahandi North Forest division | 28 | 616.17 (15.96%) | 750.16 (19.43%) | 441.66 (11.44%) | 311.81 (8.08%) | 1740.17 (45.08%) |
| Jeypur Forest Division | 29 | 8.27 (0.26%) | 38.87 (1.24%) | 68.65 (2.19%) | 263.84 (8.43%) | 2748.38 (87.86%) |
| Ghumsur South Forest Division | 30 | 817.98 (28.53%) | 435.04 (15.18%) | 363.91 (12.69%) | 431.73 (15.06%) | 817.98 (28.53%) |
| Ghumsur North Forest Division | 31 | 429.25 (25.48%) | 459.85 (27.29%) | 248.12 (14.73%) | 179.48 (10.65%) | 368.05 (21.85%) |
| Paralakhemundi Forest Division | 32 | 690.61 (17.02%) | 842.79 (20.77%) | 962.72 (23.73%) | 1262.95 (31.13%) | 298.57 (7.36%) |
| Dhenkanal Forest Division | 33 | 857.68 (19.08%) | 593.84 (13.21%) | 501.21 (11.15%) | 719.56 (16%) | 1823.7 (40.56%) |
| Deogarh Forest Division | 34 | 1273.7 (33.56%) | 876.7 (23.1%) | 498.73 (13.14%) | 510.31 (13.45%) | 636.02 (16.76%) |
| Cuttack Forest Division | 35 | 220 (4.8%) | 114.96 (2.51%) | 101.73 (2.22%) | 183.61 (4.01%) | 3961.7 (86.46%) |
| City Forest Division | 36 | 64.51 (3.48%) | 28.12 (1.52%) | 23.16 (1.25%) | 49.62 (2.68%) | 1688.06 (91.08%) |
| Chandaka Wildlife Division | 37 | 167.07 (35.19%) | 76.09 (16.03%) | 43.01 (9.06%) | 60.38 (12.72%) | 128.2 (27%) |
| Boudh Forest Division | 38 | 815.5 (27.07%) | 498.73 (16.56%) | 207.6 (6.89%) | 221.66 (7.36%) | 1268.74 (42.12%) |
| Bonai Forest Division | 39 | 1257.98 (46.67%) | 603.77 (22.4%) | 352.33 (13.07%) | 239.03 (8.87%) | 242.33 (8.99%) |
| Bolangir Forest Division | 40 | 351.51 (5.34%) | 621.13 (9.44%) | 717.08 (10.9%) | 1463.93 (22.25%) | 3424.93 (52.06%) |
| Bhadrak Wildlife Division | 41 | 0 (0%) | 0 (0%) | 0.83 (0.03%) | 19.02 (0.79%) | 2373.71 (99.17%) |
| Berhampur Forest Division | 42 | 362.26 (10.02%) | 258.05 (7.14%) | 344.06 (9.51%) | 556.62 (15.39%) | 2094.99 (57.94%) |
| Baripada Forest Division | 43 | 571.51 (12.43%) | 577.3 (12.56%) | 696.4 (15.15%) | 1197.61 (26.05%) | 1554.08 (33.81%) |
| Baragarh Forest Division | 44 | 178.65 (3.34%) | 354.82 (6.63%) | 330 (6.17%) | 727.83 (13.6%) | 3759.89 (70.26%) |
| Bamra Wildlife Division | 45 | 1061.97 (42.43%) | 485.49 (19.4%) | 256.39 (10.24%) | 342.41 (13.68%) | 356.47 (14.24%) |
| Balliguda Forest Division | 46 | 550.01 (12.27%) | 880.01 (19.63%) | 1108.28 (24.73%) | 1745.96 (38.96%) | 197.67 (4.41%) |
| Balasore Wildlife Division | 47 | 259.7 (7.14%) | 93.46 (2.57%) | 94.29 (2.59%) | 239.85 (6.59%) | 2950.18 (81.11%) |
| Athamallik Forest Division | 48 | 589.71 (31.97%) | 442.49 (23.99%) | 218.35 (11.84%) | 210.08 (11.39%) | 383.76 (20.81%) |
| Athagarh Forest Division | 49 | 439.18 (28.44%) | 167.9 (10.87%) | 150.53 (9.75%) | 171.2 (11.09%) | 615.35 (39.85%) |
| Angul Forest Division | 50 | 359.78 (12.81%) | 282.03 (10.04%) | 336.62 (11.99%) | 572.34 (20.38%) | 1257.16 (44.77%) |

Percentages represent the ratio of area under habitat suitability categories and the total area of the forest division.

*Supp. Table 6: Potential habitat use by Asian elephants overlaying with current established protected areas in the state of Odisha, India*

| Protected areas | Highly potential | Moderate highly potential | Moderately potential | Moderate least potential | Least potential |
| --- | --- | --- | --- | --- | --- |
| Badrama WLS | 247.3 (71.87%) | 90.98 (26.44%) | 5.79 (1.68%) | 0 (0%) | 0 (0%) |
| Balukhand WLS | 0 (0%) | 0 (0%) | 0 (0%) | 0 (0%) | 19.02 (100%) |
| Bhitarkanika NP | 0 (0%) | 0 (0%) | 0.83 (0.15%) | 1.65 (0.3%) | 548.35 (99.55%) |
| Chandaka WLS | 98.42 (51.07%) | 50.45 (26.18%) | 11.58 (6.01%) | 18.2 (9.44%) | 14.06 (7.3%) |
| Debrigarh WLS | 135.64 (40.39%) | 148.05 (44.09%) | 41.35 (12.31%) | 5.79 (1.72%) | 4.96 (1.48%) |
| Hadagarh WLS | 86.84 (49.3%) | 43.01 (24.42%) | 15.71 (8.92%) | 11.58 (6.57%) | 19.02 (10.8%) |
| Kapilash WLS | 98.42 (79.87%) | 19.02 (15.43%) | 2.48 (2.01%) | 0.83 (0.67%) | 2.48 (2.01%) |
| Karlapat WLS | 127.37 (63.9%) | 66.99 (33.61%) | 4.96 (2.49%) | 0 (0%) | 0 (0%) |
| Khalasuni WLS | 103.38 (86.81%) | 15.71 (13.19%) | 0 (0%) | 0 (0%) | 0 (0%) |
| Kotagarh WLS | 114.96 (22.71%) | 156.32 (30.88%) | 145.57 (28.76%) | 89.32 (17.65%) | 0 (0%) |
| Kuldiha WLS | 196.84 (75.08%) | 43.01 (16.41%) | 9.92 (3.78%) | 9.92 (3.78%) | 2.48 (0.95%) |
| Lakhari Valley WLS | 56.24 (40%) | 62.03 (44.12%) | 15.71 (11.17%) | 4.14 (2.94%) | 2.48 (1.76%) |
| Satkosia TR | 525.19 (53.27%) | 269.63 (27.35%) | 72.78 (7.38%) | 52.93 (5.37%) | 65.34 (6.63%) |
| Sunabeda WLS | 103.38 (16.62%) | 315.12 (50.67%) | 138.12 (22.21%) | 52.11 (8.38%) | 13.23 (2.13%) |
| Similipal TR | 1193.47 (44.21%) | 1114.07 (41.27%) | 349.85 (12.96%) | 41.35 (1.53%) | 0.83 (0.03%) |
| Total Area | **3087.45** | **2394.39** | **814.65** | **287.82** | **692.25** |

Abbreviations: WLS: Wildlife Sanctuary; NP: National Park; TR: Tiger Reserve. Percentages are relative to the whole area of the protected area.

*Supp. Table 7: Details on division-wise length of the potential movement pathways for Asian elephants in the state of Odisha, India*

| Forest & Wildlife Divisions | Serial No. (as mentioned in Fig. 4) | Cumulative length of Elephant movement pathways in the particular division (km) |
| --- | --- | --- |
| Sundargarh Forest Division | 1 | 81.71 |
| Sunabeda Wildlife Division | 2 | 0.55 |
| Subarnapur Forest Division | 4 | 103.24 |
| Satkosia Wildlife Division | 5 | 0.75 |
| Hirakud Wildlife Division | 6 | 24.19 |
| Sambalpur South Forest Division | 7 | 324.1 |
| Sambalpur North Forest Division | 8 | 31.7 |
| Rourkela Forest Division | 9 | 55.18 |
| Rayagada Forest Division | 10 | 56.06 |
| Rairakhol Forest Division | 13 | 275.78 |
| Phulbani Forest Division | 16 | 192.91 |
| Nayagarh Forest Division | 17 | 53.51 |
| Nawarangpur Forest Division | 18 | 188.68 |
| Mahanadi Wildlife Division | 20 | 0.02 |
| Koraput Forest Division | 21 | 8.49 |
| Khurda Forest Division | 22 | 45.56 |
| Khariar Forest division | 23 | 75.59 |
| Keonjhar Wildlife Division | 24 | 105.51 |
| Keonjhar Forest Division | 25 | 343.09 |
| Karanjia Forest Division | 26 | 4.18 |
| Kalahandi South Forest Division | 27 | 281.7 |
| Kalahandi North Forest division | 28 | 226.18 |
| Jeypur Forest Division | 29 | 68.59 |
| Ghumsur South Forest Division | 30 | 192.36 |
| Ghumsur North Forest Division | 31 | 209.15 |
| Paralakhemundi Forest Division | 32 | 53.43 |
| Dhenkanal Forest Division | 33 | 286.66 |
| Deogarh Forest Division | 34 | 516.42 |
| Cuttack Forest Division | 35 | 34.23 |
| Chandaka Wildlife Division | 37 | 3.19 |
| Boudh Forest Division | 38 | 136.55 |
| Bonai Forest Division | 39 | 220.98 |
| Bolangir Forest Division | 42 | 163.06 |
| Baripada Forest Division | 43 | 13.52 |
| Baragarh Forest Division | 44 | 158.44 |
| Bamra Wildlife Division | 45 | 194.93 |
| Balliguda Forest Division | 46 | 80.41 |
| Balasore Wildlife Division | 47 | 0.03 |
| Athamallik Forest Division | 48 | 113.55 |
| Athagarh Forest Division | 49 | 101.93 |
| Angul Forest Division | 50 | 134.3 |
